# Root Canal Treatment of a Maxillary Second Premolar with Two Palatal Root Canals: A Case Report

**DOI:** 10.7508/iej.2016.03.017

**Published:** 2016-05-01

**Authors:** Maryam Golmohammadi, Hamid Jafarzadeh

**Affiliations:** a *Endodontic Department, Dental School, Mashhad University of Medical Sciences, Mashhad, Iran*

**Keywords:** Anatomical Variations, Maxillary Second Premolar, Root Canal Therapy

## Abstract

Accurate diagnosis of the root canal morphology and anatomy is essential for thorough shaping and cleaning of the entire root canal system and consequent successful treatment. This report describes a case of maxillary second premolar with two roots and three root canals (two mesial and distal palatal canals). The case report underlines the importance of complete knowledge about root canal morphology and possible variations, coupled with clinical and radiographic examination in order to increase the ability of clinicians to treat difficult cases.

## Introduction

Root canal system has a wide morphological divergence [1]. Success of endodontic treatment depends on the knowledge of root canal anatomy. This is especially essential in cases where extra root canals are expected [2, 3]. Proper care and attention must be exercised in detection and negotiation of extra root(s)/canal(s) [4]. 

Endodontic treatment of maxillary second premolars offers a clinical challenge because of the vast anatomical and morphological variations among different racial and ethnic groups [5]. Despite being rare, the possibility of extra roots or canals should always be considered to ensure successful endodontic treatment [6, 7]. The literature reveals wide variations in root canal morphology of maxillary premolars. Pineda and Kuttler [8], did not report any three rooted maxillary second premolars. Vertucci [9] reported an incidence of 1% for maxillary second premolars with three canals while Pecora *et al. *[10] mentioned this to be 0.3%. Three rooted maxillary premolars look anatomically similar to the molars and are sometimes called small/mini molars or radiculous [11, 12].

According to the literature, three-rooted maxillary premolars usually have a mesiobuccal, a distobuccal and a palatal canal [13]. However, the present case report describes the treatment challenges of a two-rooted maxillary second premolar with one buccal root and two palatal canals within a single palatal root.

## Case Report

**Figure 1:**
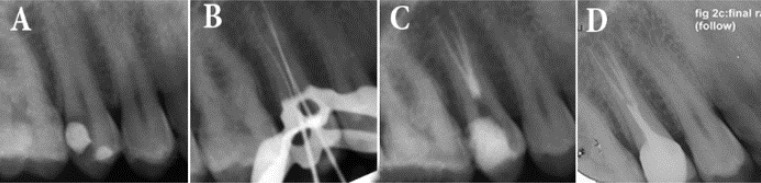
*A)* Initial periapical radiography; *B)* Working radiography showing three files in three canals; *C)* Final radiography and CBCT; *D)* Follow-up radiography after six months

A 30-year old male with non-contributory medical history came to the dental clinic of the Faculty of Dentistry, Mashhad University of Medical Sciences, Mashhad, Iran, complaining of pain in upper right posterior region. On clinical examination, an old composite restoration was seen on the maxillary right second premolar. The tooth was sensitive to cold and electric pulp testing (Analytic Technology, Redmond, WA, USA) indicating irreversible pulp inflammation. The periapical region appeared radiographically normal and uncommon root configuration was evident (Figure 1A). The tooth was not sensitive to percussion and palpation.

Access opening was done under local anesthesia (2% Lidocaine plus epinephrine 1:80000) (Daroupaksh, Tehran, Iran), after rubber dam isolation. The unusual anatomy made it difficult to precisely discern, whether the roots were located on the buccal or palatal aspect. The T-shaped access cavity was modified as described by Balleri *et al.* [14]; with the cavity preparation starting at the bucco-proximal angle from the entrance of the buccal canal to the cavosurface angle resulting in a T-shaped outline. But only one buccal and one palatal orifice could be located. In the floor of pulp chamber, only two orifices were detected. Even with the exploration of the access cavity, no other orifices were found. Using two #15 K-files (Mani, Tochigi, Japan) the working length was determined radiographically. This radiography revealed two canals with a single outline within the palatal root. A #15 K-file with obtuse precurve in the apical third was placed alongside to the palatal and a radiography was taken which confirmed the division of palatal canal into two mesial and distal separate canals in the coronal third (Figure 1B). Working length of the extra canal was determined radiographically and confirmed using an apex locator (iPex, NSK, Nakanishi Inc., Tokyo, Japan). Canals were prepared using 25/0.06 Mtwo rotary files (VDW, Munich, Germany) and 5.25% NaOCl irrigation. Canals were dried with sterile paper points and obturated with cold lateral condensation technique using AH 26 sealer (De Trey, Dentsply, Konstanz, Germany). Final radiography was taken to confirm the quality of the obturation (Figure 1C) and the tooth was restored with full crown. A six-month recall radiography and absence of any symptoms indicated satisfactory treatment outcome (Figure 1D).

## Discussion

The present case report discussed the endodontic treatment of a maxillary premolar with two palatal canals. Despite being rare, this anatomy may be encountered during routine endodontic treatments; so practitioners should be able to manage these complicated cases. Unidentified root canals represent a major cause of endodontic treatment failure.

Endodontic success in teeth with more-than-expected canals, requires a correct diagnosis and careful clinical and radiographic inspection. Morphological variations in pulpal anatomy must be always considered before beginning treatment [15].

Maxillary premolars with three root canals are sometimes called small/mini molars or radiculous because of their similar anatomy to that of adjacent maxillary molars [11, 12]. In straight-on radiographies of maxillary premolars, Sieraski *et al.* [13] found that whenever the mesio-distal width of the mid-root was equal to or greater than the mesio-distal width of the crown, the tooth is likely to have three roots. 

The access cavity for maxillary second premolars is usually oval [16] with the greater diameter being located in the bucco-palatal direction. In three-rooted maxillary premolars, the buccal orifices are usually close to each other and are hard to locate. Balleri *et al. *[17] suggested a T-shaped access outline for three rooted maxillary first premolars. This modification allows good access to the two buccal canals. But in this case a further modification had to be done as the canals were located on the palatal aspect of the tooth. Palatal canal was bifurcated in the coronal third region and thus showed two mesial and distal foramina. The possible anatomic configurations of maxillary premolars are well documented in the literature. High quality preoperative radiographies and their careful examination are essential for the detection of additional root canals [18-20]. However, radiographies are two-dimensional images of a three-dimensional objects. The clinician must be aware of these limitations during radiographic interpretation [21, 22].

In today's endodontic practice aberrant anatomy has become more common than before and, therefore, clinicians should be alert for variations in anatomy as the successful outcome of any treatment depends on the complete debridement and disinfection of all canals.

## Conclusion

Morphological variations in the pulpal anatomy must be always considered before beginning treatment. Clinicians should be aware of anatomical variations in maxillary premolars and be able to apply the knowledge in radiographic and clinical interpretation.
